# Mesoporous Materials: Materials, Technological, and Environmental Applications

**DOI:** 10.3390/ijms24119197

**Published:** 2023-05-24

**Authors:** Izabela Nowak, Agnieszka Feliczak-Guzik

**Affiliations:** Faculty of Chemistry, Adam Mickiewicz University in Poznań, Uniwersytetu Poznańskiego 8, 61-614 Poznań, Poland; nowakiza@amu.edu.pl

Research on the synthesis and characterization of ordered mesoporous materials with uniquely functionalized external and internal surfaces has intensified in the last decade. Ordered mesoporous materials consist of mesopores of variable size and regular shape, and can be considered crystalline materials in terms of organized porosity, even if they are built of amorphous walls. Therefore, a key step to understanding their properties is the structural and textural analyses of mesoporous materials using basic techniques such as X-ray diffraction (XRD or SAXS), optical spectroscopy (e.g., infra-red), electron microscopy (e.g., transmission and nitrogen sorption analysis), and more advanced ones, for example, Raman spectroscopy, MALDI TOF MS, ^13^C solid CP-MAS NMR, or thermic analysis (e.g., thermogravimetry).

Ordered mesoporous materials comprise various types of materials, including silicas, organosilicacompounds, metal oxides, sulfides, nitrides, carbonitriles and nitrides, pure metals, or zeolites, among others. It is worth noting that current synthetic strategies make it possible to obtain materials with combined properties, such as those associated with ordered mesoporous structures and those typical of specific base materials (e.g., oxides, metals, or zeolites). Systems synthesized in this way exhibit new properties for future material, technological, and environmental applications, ranging from adsorption, catalysis, electrochemistry, (bio)sensors, optics, and energy storage, to pharmaceutical and bioapplications [1,2,3,4,5].

This Special Issue titled “Mesoporous Materials: Materials, Technological and Environmental Applications” of the *International Journal of Molecular Sciences* focuses on recent developments in the synthesis, functionalization, and application of ordered mesoporous materials. It includes a total of five original articles providing new information on the synthesis, functionalization, and potential application of porous materials.

Hierarchical zeolites are an example of materials that are attracting increasing scientific interest. These are materials with at least two pore systems, i.e., always micropores (less than 2 nm in diameter) and meso- or macropores (more than 2 nm or 50 nm in diameter, respectively) [6]. The contributions and properties of secondary porosity (specific surface area, pore size and distribution, and pore volume) depend primarily on the method of synthesis of hierarchical zeolites. The introduction of secondary porosity improves the accessibility of pores for larger reactant molecules and increases the rate of diffusion. In addition, this porosity provides an ideal space for the deposition of active catalytic phases, i.e., metals, metal oxides, sulfides, nitrides, etc. [7].

In the paper by Chudzińska and co-workers [8], hierarchical zeolites and diatomaceous biosilica modified with ruthenium and silver ions were used for the first time as catalysts for the photocatalytic removal of bisphenol A. This organic chemical is widely used, among others, in the production of epoxy resins, plastics, and thermal receipt papers. Despite its numerous applications, bisphenol A exhibits negative effects on human health and this contributed to the search for an effective method for its removal. The authors optimized the process conditions, i.e., temperature, pH, and composition of the reaction mixture and electromagnetic wavelength of the light source. All of the materials obtained were characterized using appropriate techniques, such as X-ray diffraction and low-temperature nitrogen adsorption/desorption isotherms. Of all the catalysts used, ruthenium ion-modified biosilica proved to be the most effective material for bisphenol A removal, with removal rates of more than 99%.

The use of biosilica doped with palladium(II) chloride nanoparticles in the photocatalytic degradation of the azo dye, namely methyl orange (MO), in an aqueous solution under UV light was described by Sprynskyy and co-workers [9]. The synthesized material was characterized using X-ray diffraction, TEM, and nitrogen adsorption/desorption isotherms. The pH of the methyl orange solution, temperature of the process, time of UV irradiation, and initial concentration of MO were modified during the degradation process. The authors found that efficient photodegradation of methyl orange occurred within the first minute of conducting the process with an efficiency of 85%, while after 75 min an efficiency of more than 98% was obtained.

To remove hair dyes (Arianor Madder Red 306003, Arian or Straw Yellow 306005, and Arianor Ebony 306020) from aqueous solutions in batch mode, Al-Ma’abreh et al. [10] used ZnO-coated oak dome powder (COZ). This material was characterized using X-ray diffraction (XRD), FT-IR spectroscopy, and SEM scanning electron microscopy. The authors of the paper optimized the efficiency of the adsorption process by varying the adsorbent dosage, initial concentration, contact time, temperature, and pH. The following conditions proved to be optimal for the adsorption of the above-mentioned hair dyes: adsorbent mass of 0.07 g, 0.06 g, and 0.05 g for Arianor Madder red 306003, Arian or Straw Yellow 306005, and Arianor Ebony 306020, respectively, and contact time of 120 min for Arianor Madder red 306003, and Arianor Ebony 306020, and 150 min for Straw Yellow 306005. The equally fast adsorption kinetics and high adsorption values indicate that the ZnO-coated oak domes (COZ) material is a highly competitive adsorbent for removing hair dyes from solutions.

Shen and co-workers [11] obtained mesoporous polymers (DES@MIPs) with deep eutectic solvents (α-methylacrylic acid and choline chloride) for the purification of gallic acid from *Camellia* spp. fruit peels. The obtained polymers were characterized by scanning electron microscopy, particle size analysis, nitrogen sorption porosimetry, elemental analysis, Fourier transform infrared spectroscopy, and thermogravimetric analysis. In addition, the authors focused on evaluating the obtained materials for their ability to recognize gallic acid in the fruit peel of *Camellia* spp. by determining the adsorption capacity of gallic acid to the obtained materials. The purification recovery of gallic acid from various *Camellia* spp. fruit peels ranged from 87.85% to 96.75% with a purity of more than 80%, which means that the obtained polymers are effective adsorbents and are universally applicable for the separation of target substances from complex systems.

Nowadays, the real challenge for researchers is to find materials (carriers) that would effectively and efficiently influence the bioavailability of active substances by increasing their penetration into the relevant tissues. The binding of the active substance to the carrier is supposed to influence the enhancement of the desired physicochemical characteristics of the active substances applied while weakening their undesirable properties [12,13]. Kim and colleagues [14] reported the use of mesoporous silica nanoparticles (MSNPs) modified with triphenylphosphonium cation (TPP^+^) to improve the algicidal activity of cyclohexyl-(3,4-dichlorobenzyl)amine (DP92). The authors evaluated the algicidal activity by assessing the growth inhibition of two Harmful Algal Blooms (HABs), *Heterosigma akashiwo* and *Heterocapsa circularisquama*, after treating them with mesoporous silica nanoparticles loaded with TPP or an algicide. Kim’s group results indicated that the materials used were effective in enhancing the algicidal activity of DP92, which implies the prospect of using TPP-MSNP with DP92 as an effective agent for algal removal (HAB).

To conclude, properties of porous materials such as specific surface area and pore size can be adjusted already during their synthesis. This makes it possible, among other things, to functionalize the surface with almost any type of functionalities that can be incorporated into the structure of the silica material. The different structural parameters of porous materials affect the wide possibilities of applications of these materials in various industries, such as the automotive, cosmetic, or pharmaceutical industries.
